# From shared reading to self-narration: a developmental pathway for reading-based psychological support

**DOI:** 10.3389/fpubh.2026.1982716

**Published:** 2026-10-12

**Authors:** Mengjing Zhao, Yishu Zhang

**Affiliations:** 1 Zhejiang College of Security Technology, Wenzhou, Zhejiang, China; 2 Wenzhou Medical University, Wenzhou, Zhejiang, China

**Keywords:** bibliotherapy, developmental psychology, emerging adulthood, mental health promotion, narrative identity, self-narration, shared reading

## Abstract

Reading-based psychological support is usually studied as clinical bibliotherapy, shared reading, or literacy-related intervention, and these strands are rarely followed across childhood, adolescence, and emerging adulthood. This perspective asks whether shared reading can become a source of self-narration. In childhood, reading with a responsive adult may support emotional language and meaning-making. During adolescence, some narrative resources may be selectively taken up and revised. In emerging adulthood, they may be used to organize transitions, commitments, and possible futures into an evolving life story. The account presents this as a developmental hypothesis rather than as evidence that childhood reading causes later narrative identity. Reading is treated as a complement to mental-health treatment, not as a substitute. The result is a set of empirical questions about mediation, developmental timing, relational quality, and contextual confounding.

## 1 Introduction

Reading-based psychological support appears in two largely separate literatures. Clinical bibliotherapy uses selected books to support symptom management, cognitive change, or self-guided psychological treatment (1, 2). In developmental and educational settings, shared reading is associated with language development, emotion understanding, and social interaction (3–5). The two strands are seldom considered within one developmental account, so reading tends to be framed as an age-specific intervention, a literacy practice, or a group activity rather than as a psychological resource whose function changes over time.

Reading-based psychological support is used here as an umbrella term covering these strands, not as a synonym for bibliotherapy. Clinical bibliotherapy usually begins with a psychological difficulty, developmental shared reading concerns ordinary social–emotional growth, and literacy interventions primarily target language and reading skills. What the strands share is reading encounters that help people interpret emotion, relationships, and personal change. Narrative identity is a related but distinct construct, an evolving life story that organizes past experience, present commitments, and anticipated futures (6). The argument treats reading as a possible developmental input to narrative identity, a question separate from using narrative identity as a clinical outcome.

Existing evidence does not show that childhood shared reading causes a more coherent narrative identity in emerging adulthood. Findings from adjacent literatures instead support a testable account in which relationally supported reading supplies emotional and narrative resources that may be internalized and later used during self-authorship. Three transitions organize the account: relational scaffolding in childhood, narrative internalization across adolescence, and self-narration during emerging adulthood.

The model builds on process accounts of narrative self-development (7, 8) by asking whether the text-mediated, revisitable object in shared reading adds a distinctive developmental input beyond situated storytelling and autobiographical reasoning. The contribution is to specify when and how reading may become one source of narrative material within a broader social-developmental system.

The account is compatible with clinical bibliotherapy, dialogic reading, narrative identity research, and community shared-reading practice without merging them into one intervention. Reading is not a substitute for assessment, psychotherapy, or medication; the preventive question is whether reading environments contribute to psychological resources before, between, or alongside formal treatment.

## 2 From reading as intervention to reading as developmental resource

Bibliotherapy has long carried a tension between treatment and growth. Clinical approaches begin with a defined difficulty and select material for a target population. Developmental bibliotherapy uses stories and discussion to support ordinary emotional development, especially in schools and families. Its evidence base now includes recent work on digitally mediated bibliotherapy (9).

This distinction matters because a child does not encounter a story as an isolated reader. An adult selects the material, regulates the pace, explains unfamiliar experiences, notices the child’s emotional response, and connects the story to the child’s life. The book is therefore embedded in a relational event. Dialogic-reading research has primarily measured the linguistic consequences of this event, but the same interaction may also provide emotional and interpretive scaffolding (5, 10). Questions such as “Why do you think the character did that?” or “Have you ever felt like this?” invite the child to connect narrative events with mental states and personal experience.

Shared reading is more than exposure to positive messages. The child encounters an organized representation of experience, an adult helps interpret it, and the child acquires ways to talk about feelings, relationships, conflict, and change. The process resembles parent–child reminiscing but has a different structure: reminiscing organizes first-person past events, whereas shared reading offers a revisitable third-person or symbolic world. A character can provide psychological distance while the book remains a stable object for joint attention. Three features distinguish reading from other narrative activities: a text stays available for return, so the same episode can be reinterpreted later; its symbolic world allows emotional material to be approached without direct self-disclosure; and it is portable into settings where no interlocutor is present. Oral storytelling and therapeutic dialogue share the second feature only in part, and film offers the symbolic world without the reader’s control over pace and return. Whether these features produce distinct developmental effects is an empirical question, addressed in Prediction 4. Research on elaborative reminiscing (11, 12) provides an analogue for the claim, not direct evidence that shared reading produces the same outcomes.

Across development, the proposed exposure changes form while retaining a common functional core: access to a revisitable narrative object, plus opportunities to interpret it with others and, later, in dialogue with others. The model does not treat childhood co-reading as a stable cause in itself. Early interpretive support may instead contribute to later competencies that are repeatedly revised through adolescents’ own reading, peer and family conversations, and other narrative practices.

## 3 Three developmental transitions

Evidence for the three transitions is uneven. The link from shared reading to language and social–emotional outcomes in childhood rests on replicated findings; the connection between adolescent reading and documented developmental processes is not yet established; and the link from childhood reading to emerging-adult narrative identity remains theoretical. Each subsection states which level applies, and Table 1 carries the same labels.

**Table 1:** Developmental stages, proposed mechanisms, current evidence, and testable predictions.

Stage	Mechanism and role	Evidence status	Testable prediction	Key covariates	Recommended design
Childhood shared reading	Relational scaffolding, psychological distance, and joint attention to a revisitable text (predictor)	Well supported for language, interaction, and proximal social–emotional outcomes	Interpretive interaction quality predicts later narrative-related skills beyond reading frequency	Baseline language; parental education; family reading climate; parent–child relationship quality; elaborative reminiscing	Longitudinal, baseline-adjusted, with family reading climate and reminiscing covariates
Adolescence	Selective internalization; autobiographical reasoning, narrative engagement, and interpretive language (mediators)	Partially supported; the reading-specific link is not established	Autobiographical reasoning mediates the association between earlier relational reading and later narrative identity	Baseline mediator and outcome levels; family socioeconomic status; home literacy resources; language environment	Preregistered three-wave mediation with baseline mediator and outcome measures
Emerging adulthood	Self-narration; narrative identity and flexible meaning-making (outcome)	Theoretical for the reading link	Narrative-resource use predicts narrative flexibility and adaptive coping	Personality; baseline mental health; cultural context; non-reading narrative exposure	Prospective design with an equivalence test against equally responsive non-reading interaction

The table distinguishes established evidence from the cross-stage connections proposed by this perspective.

### 3.1 Childhood: shared reading as relational scaffolding

In childhood, the primary psychological resource may be the relationship organized around the text. Shared reading creates a bounded situation in which an adult and child attend to the same symbolic world. The child can approach difficult emotions indirectly through a character, while the adult can respond without requiring the child to disclose a personal problem directly. This distance can make emotional exploration more tolerable than direct questioning.

The adult’s role goes beyond explaining the correct lesson. A supportive adult can validate ambiguity, invite several interpretations, and connect the story to the child’s experience without forcing disclosure. Repeated interactions may teach the child that inner states can be named, discussed, and placed within a sequence of events. These are early components of psychological meaning-making, still far from a mature narrative identity, but they may create conditions in which later self-narration becomes easier.

Evidence in childhood is strongest for proximal outcomes: language, parent–child interaction, emotion talk, and aspects of social–emotional development (3, 13, 14). Reading-based interventions have also been associated with emotion processing in children exposed to early adversity (15). A recent systematic review suggests that early reading promotion may affect parent–child relational and health-promoting processes, although effects remain heterogeneous (16). Some longitudinal studies associate childhood reading for pleasure with later health-related behaviors, but these associations do not establish a developmental mechanism (17). Evidence for long-term identity outcomes is much thinner, and childhood shared reading is treated here as a possible foundation rather than a completed therapeutic process.

### 3.2 Adolescence: from external stories to internalized narrative resources

Adolescence is a plausible transition point because the capacity to organize events into a temporally and causally coherent life story develops during this period. Research on autobiographical reasoning and family narrative co-construction suggests that adolescents increasingly interpret personal events through themes such as agency, continuity, belonging, and change (18, 19). Longitudinal work indicates both stability and developmental change in autobiographical reasoning across this period (20). Adolescents also encounter narratives through novels, biographies, and other media; the account treats these non-reading narratives as competing or complementary contexts rather than as evidence for a reading-specific effect.

Reading may widen the range of situations and identities available for reflection. Fiction rarely offers a direct script for living; it can provide models of conflict, attachment, loss, moral choice, and transformation that readers compare with their own experience and accept, reject, or revise. Internalization is selective appropriation, not passive absorption.

Internalization is observable at three levels. At the level of content, readers spontaneously use story-derived themes, metaphors, or contrasts when describing their own experience; at the level of structure, personal narratives show greater temporal, causal, and thematic coherence; and at the level of language, descriptions of self and others contain more emotion and mental-state terms. These indicators can be scored from life-story interviews and from linguistic coding of narrative text.

The transition changes the form of the resource rather than the target of the theory. The childhood input is relationally organized reading, the adolescent process is selective appropriation and comparison, and the emerging-adult outcome is the use of narrative resources in self-interpretation. Reading-specificity becomes a testable hypothesis when the relevant comparison is interpretive reading against equally responsive non-reading narrative interaction.

The relational mechanism changes during adolescence but does not disappear. Reading may become more solitary, while interpretations continue to be shaped in classrooms, peer groups, families, online communities, and therapeutic conversations. The shift is from overt co-reading toward an internalized dialogue that remains open to social revision.

Replication findings on fiction reading, empathy, and theory of mind are mixed (21, 22), and associations between reading and well-being may reflect family resources, education, personality, and prior mental health. Reading does not automatically improve social cognition or produce a coherent self. The claim is narrower: reading supplies symbolic material that may become useful when combined with reflection, discussion, and developmental readiness.

Three intermediate processes deserve distinction. Autobiographical reasoning is the capacity to draw temporal, causal, and thematic links among personal events. Narrative engagement is sustained interpretive involvement with a story, including attention to characters’ goals, conflicts, and possible meanings; it is not equivalent to reading frequency or passive exposure. Emotionally meaningful interpretive language is the ability to name and discuss feelings, intentions, relationships, and change in ways connected to personal experience. The processes overlap, but each does distinct work: autobiographical organization, engagement with narrative material, and the language available for interpretation.

The three processes occupy the mediator position, between relational reading experiences and narrative identity. Self-narration names the ongoing process of telling, and narrative identity names the structured product it yields, which serves as the outcome. Autobiographical reasoning, narrative engagement, and interpretive language are the intermediate processes through which earlier reading is expected to affect that outcome.

Each construct can be operationalized with existing instruments. Narrative identity is assessed with the life-story interview and scored for the Big Three themes of agency, communion, and redemption (23–25). Autobiographical reasoning is scored from turning-point narratives for meaning-making (26) and from narrative coherence ratings (27). Reading motivation and the amount and breadth of reading can be indexed with the Motivation for Reading Questionnaire (28), which serves as a behavioral proxy for narrative engagement, and emotionally meaningful interpretive language is indexed by emotion and mental-state vocabulary.

### 3.3 Emerging adulthood: self-narration and life authorship

Emerging adulthood is characterized by identity exploration, instability, self-focus, a sense of being between stages, and heightened perceived possibilities (29). These features create a demand for self-interpretation. Individuals must make choices about education, work, relationships, values, and future direction while many social roles remain unsettled.

Narrative identity theory describes emerging adulthood as a period in which individuals increasingly author their own life stories (6, 30). Recent longitudinal research has connected narrative identity development from adolescence into emerging adulthood with well-being (31), while longer-term work has linked narrative features to trajectories of depression and well-being beyond personality traits (32). Self-narration here does not mean producing a polished autobiography; it is the ongoing organization of remembered events, present commitments, and imagined futures into a personally meaningful account. Literary and biographical texts may contribute concepts, contrasts, metaphors, and possible selves for interpreting these transitions.

Shared reading can make meaning-making relationally available in childhood. Adolescence may expand the capacity to compare and internalize narrative resources. Emerging adulthood increases the need to use such resources while making choices and interpreting change. The sequence is not universal or linear: some people develop self-narrative through family stories, religion, peer relationships, media, or creative practice rather than reading.

## 4 The proposed developmental pathway

Figure 1 summarizes the proposed sequence. In childhood, children encounter stories within supportive relationships, most often through shared reading, oral storytelling, or guided discussion, and adults or peers connect narrative events with emotions, intentions, and personal experience. Across later childhood and adolescence, some stories and interpretive patterns may be internalized as symbolic resources for autobiographical reasoning. By emerging adulthood, individuals may draw on them to make sense of transitions, conflicts, commitments, and possible futures.

**Figure 1:**
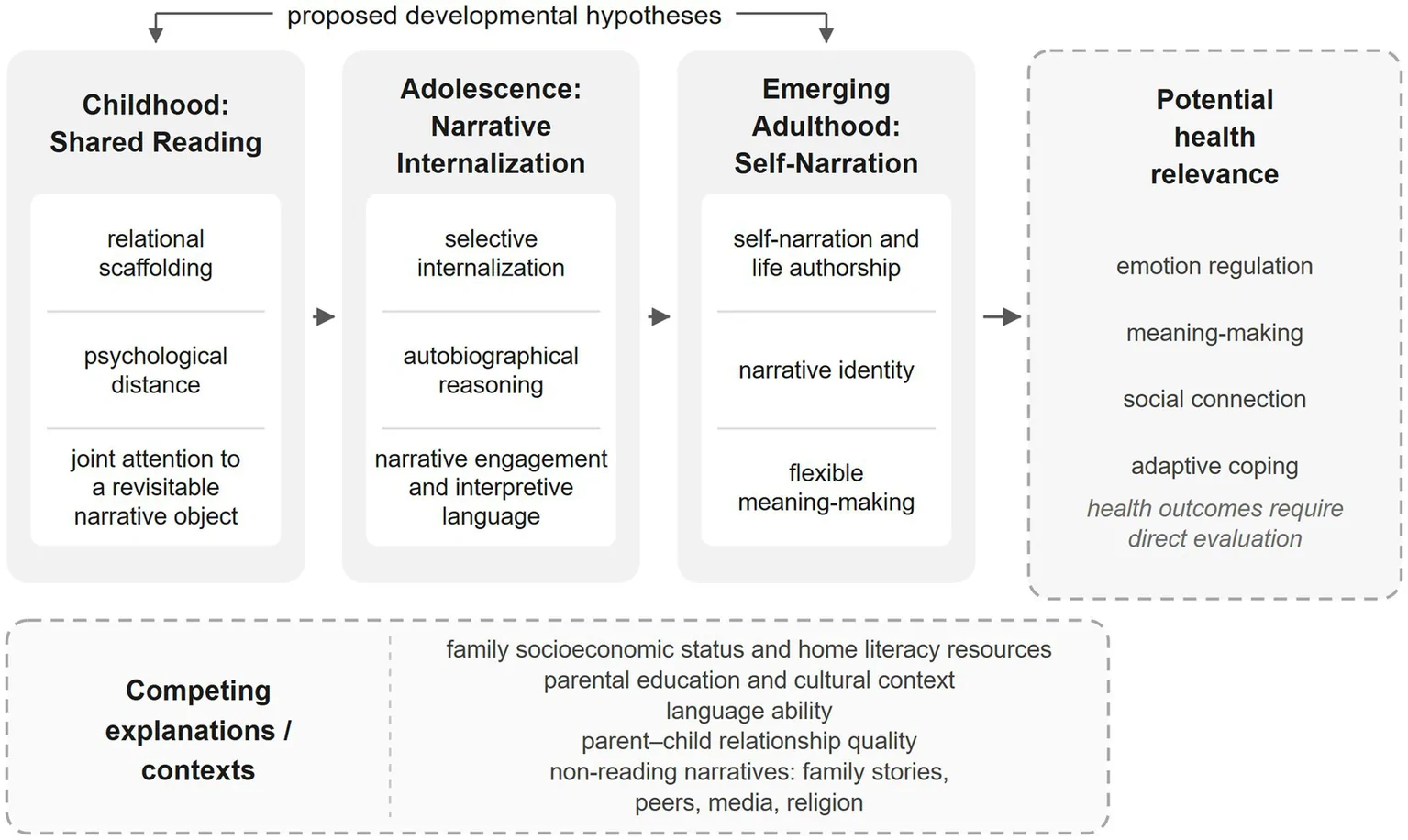
Proposed developmental mechanisms from shared reading to self-narration, with competing explanations. Solid arrows indicate hypothesized links rather than established causal effects. Dashed boxes indicate competing explanations and contexts that could account for the same outcomes without a reading-specific input, and the provisional status of the health-relevance component. Evidence status for each link is reported in Table 1.

The arrows in Figure 1 are hypotheses rather than established causal effects, and the four predictions are summarized in Table 1. The first asks whether interpretive shared reading explains additional variance in later autobiographical reasoning once baseline language, parental education, family reading climate, relationship quality, and elaborative reminiscing are accounted for. The second asks whether autobiographical reasoning temporally mediates the association between earlier relational reading and emerging-adult narrative identity. The third distinguishes frequent reading from frequent reading embedded in responsive dialogue. The fourth tests whether equally high-quality non-reading narrative interaction produces the same associations, in which case the account would broaden to relational narrative scaffolding.

The predictions are intended as empirical discriminations, not as claims that a three-wave study would establish causality. Predictions 1 and 2 concern incremental association and a longitudinal indirect pathway under explicit assumptions about baseline measures and time-varying confounding; Predictions 3 and 4 require a preregistered comparison of reading frequency against interpretive interaction quality, and an adequately powered equivalence test with prespecified bounds for non-reading narrative interaction.

The pathway may also be discontinuous. Extensive shared reading in childhood may yield few narrative resources when the interaction is coercive, emotionally unsafe, or disconnected from the child’s experience. Conversely, an adolescent with little childhood reading may acquire such resources through later literary encounters, therapy, friendship, or cultural participation. Developmental pathways are probabilistic and context-sensitive.

The model is best understood as a developmental cascade with reciprocal and competing influences rather than a one-way pipeline. Child characteristics and family resources may shape the opportunity for shared reading; later reading choices and social contexts may reshape the same competencies. Repeated measures of reading interaction, autobiographical reasoning, and narrative engagement would be needed to distinguish stable individual differences from within-person developmental change.

## 5 Psychological health implications

This framework places reading-based psychological support between universal prevention and formal treatment. It does not show that shared reading prevents, alleviates, or treats a mental disorder; the defensible claim is narrower, that relational reading may create opportunities for emotional language, reflective dialogue, and meaning-making, with downstream health effects requiring direct evaluation.

Narrative meaning-making offers one possible bridge to health psychology. A developing life story can organize stressful events, preserve continuity across transitions, and connect present choices to valued futures. These processes may support coping, but narrative coherence is not equivalent to psychological health: a coherent story may also be rigid or self-blaming. The health-relevant question is whether reading-supported narrative resources increase flexible meaning-making, emotion regulation, and social integration (23, 33). Recent realist work suggests that reading for pleasure may operate through conversation, shared meaning-making, perspective-taking, and temporary relief from stress rather than through a universal reading effect (34, 35).

The public health relevance lies in reach and cost rather than in treatment effect. Shared reading is already embedded in schools, libraries, and community programmes, and it reaches families that do not enter clinical services without depending on clinical personnel. If the developmental links proposed here hold even partially, reading environments become a modifiable, low-cost target for mental health promotion, while effects on diagnosable disorder remain untested.

Access to reading is socially graded. Family income, parental literacy, home book density, school resources, digital access, and the presence of a reading adult shape how much interpretive interaction a child receives, and these conditions also predict mental health. Apparent reading effects may therefore reflect family advantage, and reading promotion may widen gaps if it reaches only families that are already resourced. Cultural context operates similarly. The model assumes joint reading between a child and an adult, a practice that is not universal. Where oral traditions are strong, narrative resources may be transmitted through storytelling, song, or religious practice rather than print; where meaning-making is carried by the family or group rather than the individual narrator, the outcome may need to be measured at that level; and where literacy access is limited, audio, pictorial, or community formats carry the same functions. Cultural and socioeconomic variables are therefore necessary moderators rather than background controls.

In families and schools, the immediate goal is to strengthen opportunities for reflective dialogue rather than to diagnose or treat a disorder. In libraries and community settings, shared reading may create opportunities for social connection without requiring a clinical identity; whether this reduces loneliness or improves mental health requires direct evaluation. In clinical contexts, reading can serve as an adjunctive resource when chosen collaboratively and when the limits of bibliotherapy are made explicit.

This account also discourages premature medicalization. A book is not a medication, and a reading group is not automatically psychotherapy. The psychological value of reading depends on context, developmental fit, relational safety, accessibility, and the reader’s interpretation. Treating reading as a developmental resource preserves its cultural and relational dimensions while allowing its health implications to be studied with methodological caution.

## 6 Limitations and research agenda

The central limitation is the absence of direct longitudinal evidence connecting childhood shared reading to narrative identity in emerging adulthood. Existing studies support separate segments of the account, but the full pathway remains a theoretical proposition. Future research should ask whether shared reading predicts later narrative identity, whether narrative identity mediates associations between reading experiences and well-being, and whether these associations remain after accounting for family socioeconomic status, parental education, home literacy resources, language environment, cultural context, personality, and baseline mental health.

One feasible first step is a preregistered measurement and association study of emerging adults combining retrospective measures of childhood shared reading, current reading engagement, autobiographical reasoning, narrative identity, and mental-health indicators. Such a design can assess construct relations and feasibility but cannot establish temporal order, mediation, or causality. Prospective research would need at least three waves, with childhood measures of reading interaction and baseline abilities, adolescent measures of narrative reasoning, and emerging-adult measures of narrative identity and psychological health. Family resources, parental burden, and intervention accessibility should be measured alongside print, audio, digital, and socially mediated reading.

Repeated measures in each developmental period would be preferable. A defensible longitudinal mediation test would preregister the temporal ordering, measure baseline versions of the mediator and outcome, specify a confounding-control strategy, and report sensitivity analyses. Cross-lagged or growth-based models may help separate stable between-person differences from within-person change, but no statistical model removes unmeasured confounding by itself.

Cultural context enters the agenda as a moderator rather than a control. Studies should test whether the reading-narrative association varies with the dominant narrative mode of the setting (print-based or oral), with the social unit that carries meaning-making (individual, family, or group), and with the delivery format (print, audio, pictorial, or community-based). Where literacy access is limited, format becomes a testable moderator rather than a fixed design feature.

## 7 Discussion

Reading-based psychological support has been examined as treatment, literacy practice, social participation, and identity work. A developmental account can connect these perspectives without merging them into one intervention. Shared reading may provide relational scaffolding in childhood, reading experiences may become narrative resources across adolescence, and emerging adults may use those resources while authoring an unsettled life story.

The value of this account lies in specifying what should be tested, for whom, under which relational conditions, and through which developmental mechanisms. Rather than asking whether books are therapeutic in the abstract, it asks how stories encountered with others may become part of the language through which a person later understands and narrates a life.

The more specific theoretical question is whether the revisitable and socially interpretable structure of reading adds explanatory value beyond general narrative socialization. If it does not, a developmental account of narrative scaffolding remains the appropriate contribution, with shared reading treated as one culturally accessible pathway rather than a privileged mechanism.

The framework extends process models of narrative self-development rather than replacing them. Those models locate identity work in situated storytelling and social response; the addition here is an input that precedes and accompanies the telling, since a text-mediated object gives the developing narrator material that is available before the capacity to tell is fully formed. If the reading-specific hypothesis fails, that is, if interpretive reading proves indistinguishable from equally responsive non-reading interaction, the framework reduces to a relational account of narrative scaffolding. That outcome would still be informative: it would place the active ingredient in the quality of interpretation rather than in the medium, and would redirect intervention design toward dialogue rather than book distribution.

## Data Availability

The original contributions presented in the study are included in the article/supplementary material, further inquiries can be directed to the corresponding author.
